# HepG2BD: A Novel and Versatile Cell Line with Inducible HDV Replication and Constitutive HBV Expression

**DOI:** 10.3390/v16040532

**Published:** 2024-03-29

**Authors:** Matthieu Blanchet, Léna Angelo, Yasmine Tétreault, Marwa Khabir, Camille Sureau, Andrew Vaillant, Patrick Labonté

**Affiliations:** 1 INRS-Centre Armand-Frappier Santé Biotechnologie, Laval, QC H7V 1B7, Canada; lena.angelo@inrs.ca (L.A.); yasmine.tetreault@inrs.ca (Y.T.); khabirmarwa@outlook.com (M.K.); 2 Replicor Inc., Montréal, QC H4P 2R2, Canada; availlant@replicor.com; 3 INSERM U1259, Université de Tours, 37032 Tours, France; camille.sureau@univ-tours.fr

**Keywords:** HBV, HDV, cell line, CRISPR-Cas9, antiviral

## Abstract

Individuals chronically infected with hepatitis B virus (HBV) and hepatitis Delta virus (HDV) present an increased risk of developing cirrhosis and hepatocellular carcinoma in comparison to HBV mono-infected individuals. Although HDV only replicates in individuals coinfected or superinfected with HBV, there is currently no in vitro model that can stably express both viruses simultaneously, mimicking the chronic infections seen in HBV/HDV patients. Here, we present the HepG2BD cell line as a novel in vitro culture system for long-term replication of HBV and HDV. HepG2BD cells derive from HepG2.2.15 cells in which a 2 kb HDV cDNA sequence was inserted into the adeno-associated virus safe harbor integration site 1 (AAVS1) using CRISPR-Cas9. A Tet-Off promoter was placed 5′ of the genomic HDV sequence for reliable initiation/repression of viral replication and secretion. HBV and HDV replication were then thoroughly characterized. Of note, non-dividing cells adopt a hepatocyte-like morphology associated with an increased production of both HDV and HBV virions. Finally, HDV seems to negatively interfere with HBV in this model system. Altogether, HepG2BD cells will be instrumental to evaluate, in vitro, the fundamental HBV–HDV interplay during simultaneous chronic replication as well as for antivirals screening targeting both viruses.

## 1. Introduction

Despite a safe and effective hepatitis B vaccine [1], more than 290 million people worldwide suffer from chronic hepatitis B virus (HBV) infection [2]. These individuals are at risk of developing cirrhosis and hepatocellular carcinoma (HCC). The prevalence of hepatitis Delta virus (HDV), a defective virus and an obligate satellite of HBV, was recently updated to more than 70 million cases worldwide [3]. Carriers of HBV/HDV viruses are at increased risk of cirrhosis and HCC [4,5], making it all the more important to develop more versatile in vitro models for HDV-oriented antiviral research.

Currently approved treatments for HBV/HDV coinfected patients exhibit suboptimal efficacy [6,7]. In vitro models using stable hepatoma cell lines replicating HBV [8,9,10] have been extensively used for antiviral screening. In contrast, in vitro models for HDV lifecycle studies have relied on transient co-transfections [11,12]. However, transient transfection of cloned DNA is not very efficient, short-lived with the presence of a massive amount of recombinant DNA into transfected cells. 

Models have recently been developed to study the HDV lifecycle: (i) HepNB2.7 cells stably express the HBV/HDV receptor (the sodium taurocholate co-transporting polypeptide-NTCP) and the HBV envelope proteins. These cells allow HDV replication after inoculation with HDV virions [13]. (ii) Huh7-END cells contain a greater-than-genome-length HDV cDNA integrated randomly into cellular DNA and lentiviral-integrated sequences of NTCP coding DNA as well as HBV DNA coding for the HBV envelope [14]. (iii) Huh7-2C8D cells were engineered through lentivirus integration of DNA coding for HBV envelope proteins, along with random integration of a greater-than-genome-length HDV cDNA [15]. 

Here, we present the engineering and characterization of an HBV-replicating cell line that replicates HDV RNA and produces HDV virions in a doxycycline (dox)-dependent manner. The cell line, referred to as HepG2BD, is derived from the HepG2.2.15 cell line in which an HDV genomic cDNA was inserted into chromosomal DNA. As opposed to the aforementioned cell lines [14,15], HepG2BD cells allow for inducible synthesis of genomic HDV RNA from a CRISPR-Cas9-mediated insertion of the viral sequence into the adeno-associated virus safe harbor integration site 1 (AAVS1) genome safe harbor of cellular DNA. This procedure ensures reliable and tightly induced transcription of genomic HDV RNA from the integrated DNA template upon removal of dox. The precise integration of HDV cDNA at a defined location minimizes the risk of off-target gene expression alteration [16] and cytotoxicity [17]. The HepG2BD cell line has been stable for more than 6 months in culture, during which HDV RNA could be repressed in the presence of dox, and reliably launched upon its removal. When quiescence of the HepG2BD cells was induced by long-term maintenance of cell monolayers, an increase in several logs in HDV virions secretion, concomitant with a “hepatocyte-like” morphology, was observed.

This unique cell line will allow for both screening and characterization of new therapeutics against HBV/HDV, as well as a better understanding of the interplay between the viruses and cellular host factors involved in their lifecycles.

## 2. Materials and Methods

### 2.1. Cell Culture and Reagents

Hepatoma cell lines obtained from Camille Sureau were maintained in William’s E Medium (Thermo-Fisher, St-Laurent, QC, Canada), 10% Fetal bovine serum (FBS) (Wisent, Sain Jean Baptiste, QC, Canada), 1 μg/mL gentamicin (Thermo-Fisher, St-Laurent, QC, Canada). HEK-293T cells were maintained in Dulbecco’s Modified Eagle Medium (Thermo-Fisher, St-Laurent, QC, Canada), 10% FBS, 1 μg/mL gentamicin, sodium pyruvate 1 mM. HepG2.2.15 transfections using CRISPR-Cas9-related plasmids were carried out using lipofectamine 3000 (Thermo-Fisher, St-Laurent, QC, Canada).

### 2.2. Plasmids and Cloning

CRISPR-Cas9-related plasmids pAAVS1-puro-Tet3G [18] and eSpCas9(1.1)-AAVS1-T2 [18] were purchased from Addgene. pAAVS1-SA-T2A-Puro-TetOff-gHDV was derived from pAAVS1-puro-Tet3G (Figure S1). pWPI-puro plasmid was obtained from Laurent Chatel-Chaix [19] and Gag-Pol [20] and VSV-G [20] from Addgene. pWPI-puro-NTCP was obtained by cloning the NTCP sequence from the pCI-neo-NTCP plasmid [21]. 

### 2.3. Engineering of the Huh7-NTCP(L) Cell Line

The synthesis of pseudotyped lentiviral particles was conducted by co-transfection of HEK-293T with pWPI-Puro-NTCP, Gag-Pol, and VSV-G, in the presence of polyethylenimine (PEI) (Sigma, Oakville, ON, Canada) [19]. Lentiviruses were used to inoculate Huh7 cells in the presence of polybrene. Cells were selected with 2 μg/mL puromycin (puro) (Thermo-Fisher, St-Laurent, QC, Canada) and are referred to as Huh7-NTCP(L).

### 2.4. HDV Infection Assay 

Selected inoculums were adjusted to 4% PEG-8000 (Sigma, Oakville, ON, Canada) and added to Huh7-NTCP(L) for 16 h. Cells were then washed with PBS every 2 to 3 days. At day 10 post-inoculation, cells were harvested and total cellular RNA was extracted. NTCP mRNA and total HDV RNA were quantified as described below. Total RNA was also analyzed for the presence of genomic and/or antigenomic HDV RNA by Northern blot as described below.

### 2.5. Immunoprecipitation

Benzonase-treated supernatants were complemented with 5 μL anti-preS1 antibodies (Santa-Cruz, Dallas, TX, USA #SC-57761), or 20 μL of the isotype counterpart (negative control; Santa-Cruz #SC-3878) along with 20 μL of Protein A/G PLUS-Agarose beads (Thermo-Fisher, St-Laurent, QC, Canada). The mix was incubated for 2 h at RT under rotation. Samples were washed with PBS prior to lysis and DNA extraction using the QIAamp DNA Mini Kit (Qiagen, Toronto, ON, Canada). The qPCR was conducted as described below.

### 2.6. ELISA

HBsAg in supernatant was measured by ELISA using the GS HBsAg EIA 3.0 kit (Bio-Rad, St-Laurent, QC, Canada #32591). Relative concentration calculations were carried out using a standard curve from dilution of HepG2.2.15 reference supernatant [22,23]. Absolute quantifications were estimated using the mean absorbance/cutoff ratio provided in the kit manual.

### 2.7. RT-qPCR and qPCR

Total cellular RNA was extracted using the Aurum™ Total RNA Mini Kit (Bio-Rad, St-Laurent, QC, Canada). Purified RNA was then normalized using a nanodrop. RNA from benzonase-treated supernatants was extracted using the Qiamp RNA mini kit (Qiagen, Toronto, ON, Canada). For total HDV RNA (Genomic plus Antigenomic), HBV 3.5 kb, NTCP, and albumin mRNAs quantification, reverse transcription was conducted using the iScript™ Select cDNA Synthesis Kit (Bio-Rad, St-Laurent, QC, Canada) with random hexamers as primers. Taqman qPCR from HDV reverse-transcribed RNA was conducted using the SsoAdvanced™ Universal Probe^®^ Supermix (Bio-Rad, St-Laurent, QC, Canada) using 5’-tggacgtgcgtcctcct-3′ and 5′-tcttcgggtcggcatgg-3′ primers along with a (5/6-FAM) 5′-atgcccaggtcggac-3′ (5/6 TAMRA) probe [21]. NTCP, albumin, and HBV 3.5 kb reverse-transcribed mRNAs, as well as immunoprecipitated HBV DNA (Dane particles) quantification were conducted using the SsoAdvanced™ Universal SYBR^®^ Green Supermix. Primers for HBV and NTCP were as described [21,24]. Primers for albumin were Forward 5′-gtgtttcgtcgagatgcac-3′ and reverse 5′-tcaaatggacactgctgaag-3′. Results were compared relative to a reference sample using the Δct method [25]. 

Absolute quantification of HDV RNA was achieved by RT-qPCR using a standard curve consisting in quantified and serially diluted in vitro full-length transcribed HDV genomic RNA as previously described [26]. Quantified samples are indicated in figures with the “‡” symbol, and concentrations are documented in legends.

Absolute quantification of HBV DNA was achieved by qPCR using a standard curve consisting in quantified and serially diluted pCIHBenv(−) plasmid [12]. Quantified samples are indicated in figures with the “§” symbol, and concentrations are documented in legends.

### 2.8. Western Blot

The proteins were resolved by SDS-PAGE, transferred to PVDF membranes (Bio-Rad, St-Laurent, QC, Canada), blocked and incubated with primary antibody [Mouse monoclonal anti-βactin (Sigma, Oakville, Canada) or Human polyclonal anti-HDAg [27]]. Washed membranes were incubated for 1 h at RT with a goat-anti-mouse or goat-anti-human IgG conjugated to HRP (Jackson ImmunoResearch, Baltimore, MD, USA). Protein bands were visualized with the Clarity western ECL (Bio-Rad, St-Laurent, QC, Canada) using a Sapphire Biomolecular Imager (Azure Biosystems, Montréal, QC, Canada).

### 2.9. Northern Blot

Probes synthesis: Genomic (588 nt) and antigenomic (593 nt) HDV RNA fragments were obtained by T7-transcription in presence of biotin-11-UTP (MAXIscript T7-Transcription, Thermo-Fisher, St-Laurent, QC, Canada) of PCR amplicons containing T7 promoters. Unlabeled RNAs of each polarity were produced and used as specific controls. Northern blot was conducted as follows: purified intracellular RNA (1 µg) and genomic or antigenomic controls (200 pg) were denatured 30 min at 50 °C in glyoxal loading buffer and run in a 1% agarose gel according to the NorthernMax^tm^-Gly protocol (Thermo-Fisher, St-Laurent, QC, Canada). RNAs were transferred to a positively-charged nylon membrane (Roche, Laval, QC, Canada) and UV crosslinked (1200 J). Prehybridization, hybridization, and detection were performed as per instructions (North2South; Thermo-Fisher, St-Laurent, QC, Canada). Genomic polarity was detected using 10 ng/mL of biotinylated antigenomic probe and antigenomic polarity was detected using 2.5 ng/mL of biotinylated genomic probe. Visualization was performed using a Sapphire Biomolecular Imager (Azure Biosystems, Montréal, QC, Canada).

### 2.10. Contrast Phase and Confocal Immunofluorescence Microscopy

Phase contrast pictures were taken using the Cytation™ 5. For immunofluorescence assays, cells were cultured on collagen-coated glass coverslips and fixed for 10 min in 4% paraformaldehyde. Permeabilization with PBS complemented with 0.2% TritonX-100 for 30 min was followed by incubation with blocking solution (PBS supplemented with 3% BSA and 10% FBS) for 30 min. HDAg was detected by incubation with human anti-HDAg serum for 1 h at RT, followed by incubation of 1 h at RT with Alexa Fluor™ 568 Goat anti-Human IgG diluted at 1:1000 (Thermo-Fisher, St-Laurent, QC, Canada). HBsAg was detected by incubation of cells with anti-HBsAg diluted at 1:150 (Abcam, Toronto, ON, Canada #ab9193) followed by incubation with Alexa Fluor^®^ 488 AffiniPure Goat Anti-Horse IgG diluted at 1:10,009 (Thermo-Fisher, St-Laurent, QC, Canada). Cell nuclei were stained with DAPI. Coverslips were mounted on microscope slides using Prolong antifade reagent (Thermo Fisher, St-Laurent, QC, Canada). Cells were analyzed using a confocal microscope (Zeiss LSM 780, Dorval, QC, Canada). 

### 2.11. Antiviral Treatment

HepG2BD cells were seeded at 150,000 cells/well in collagen-coated 24-well plates. At day 3 post-seeding, cells were treated with various concentrations of FXR agonist GW4064 (Sigma, Oakville, ON, Canada) or an untreated control with DMSO. Media was then replaced every 3 days and supernatant and cells were collected at day 10 post-treatment for analysis or infection of Huh7-NTCP(L) as described in Section 2.4.

### 2.12. Statistical Analysis

Statistical analyses were performed using GraphPad Prism 5.0 software. Error bars are representative of the calculated standard deviation. When deemed necessary, one-tail unpaired (homoscedastic) Student’s *t*-tests or one-way ANOVA followed by Dunnett’s multiple comparison tests were conducted. Symbols for calculated *p*-values are as follows: ns > 0.05; * ≤ 0.05; ** ≤ 0.01; *** ≤ 0.001.

## 3. Results

### 3.1. Generation of the HepG2BD Cell Line

**Figure 1:**
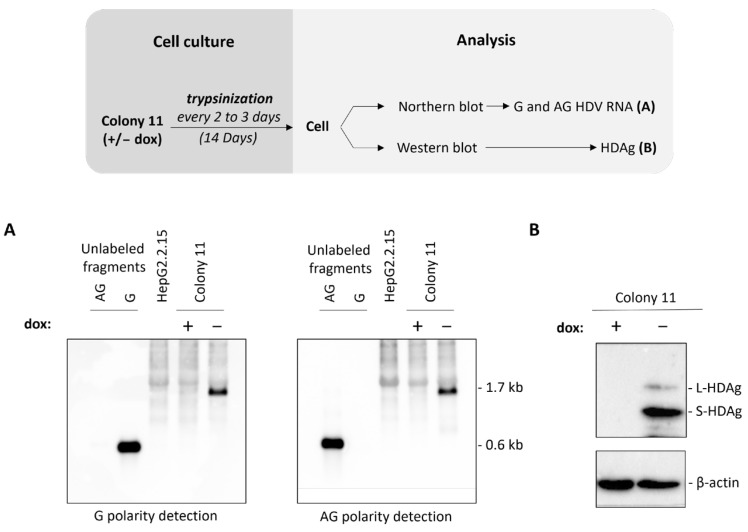
Confirmation of HDV-regulated replication in colony 11 by Northern and Western blot. Cells were cultured as depicted in the diagram for 14 days in the presence or absence of dox. (**A**) RNA was extracted from cell lysate, normalized after quantification with a nanodrop, and subjected to Northern blot for the detection of both polarities of HDV RNA in the presence or absence of dox. (**B**) Cellular total proteins were analyzed for the presence of HDAg isoforms in the presence or absence of dox by Western blot. G, genomic; AG, antigenomic.

After co-transfection with the plasmid coding for Cas9 and sgRNA [eSpCas9(1.1)/AAVS1-T2] along with the donor plasmid containing the HDV sequence [pAAVS1-SA-T2A-Puro-TetOff-gHDV] (Figure S1), several rounds of selection with puro were conducted to favor clonality. At 47 days post-selection, colonies were harvested, expanded, and tested for the expression of HDAg. Most colonies expressed HDAg, with the highest expression in colony 11 (Figure S1). Cells from this colony were treated with/without dox for 14 days as depicted in Figure 1. Both genomic and antigenomic HDV RNAs were detected by Northern blot (Figure 1A). The presence of antigenomic HDV RNA, an intermediate of replication, as well as the detection of L-HDAg, (Figure 1B) are proof of viral RNA replication. No signal was observed in the presence of dox (Figure 1A,B). In colony 11, proper chromosomal integration of the HDV cDNA sequence in the AAVS1 safe harbor was assessed (Figure S2).

Cells were passaged using 0.5 μg/mL puro for more than 3 months to maximize clonality. The resulting cell line was named HepG2BD.

### 3.2. HepG2BD Cell Line Allows Constitutive Expression of HBV and Inducible Replication of HDV

**Figure 2:**
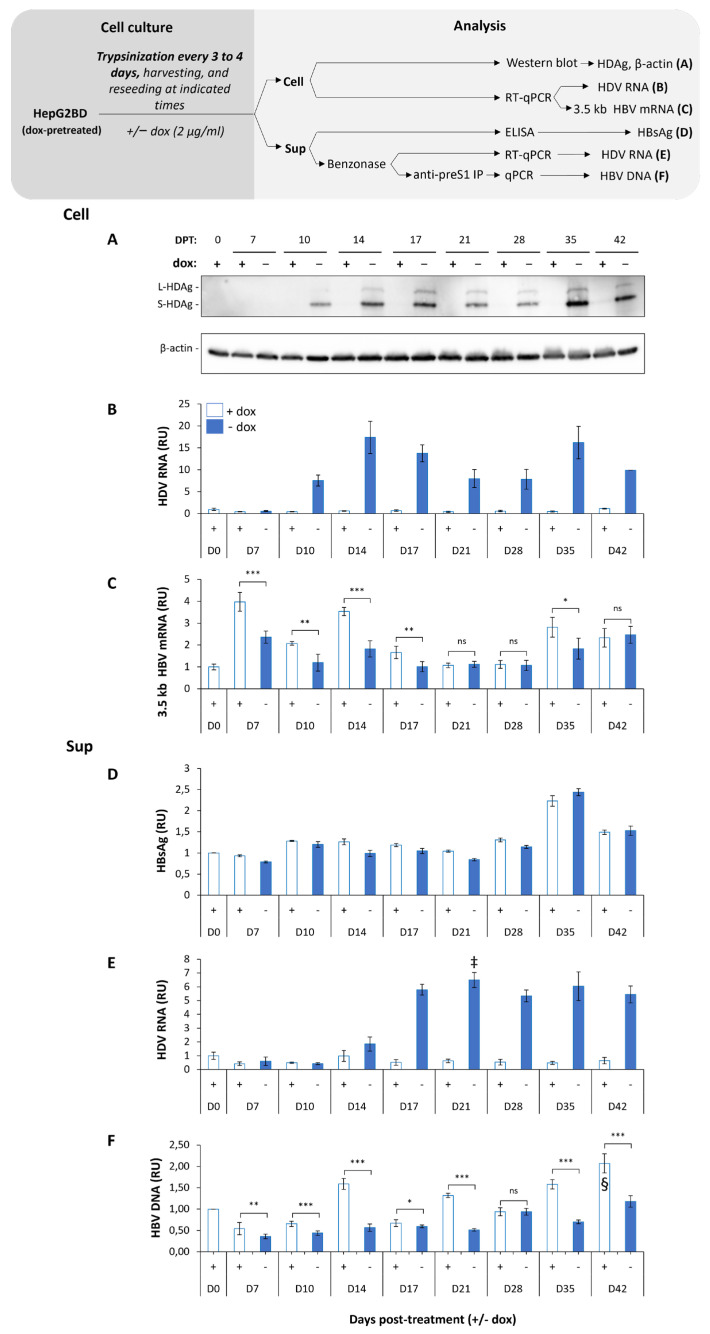
Characterization of HepG2BD cells. Dox pre-treated cells were seeded and cultured in the presence or absence of dox as depicted in the diagram. Cells and supernatants were harvested at the indicated days post-treatment (DPT). Cells were analyzed for the presence of HDAg isoforms normalized to β-actin (**A**), HDV RNA (**B**), and HBV 3.5 kb mRNA (**C**). Supernatants were analyzed for the presence of HBsAg (**D**), HDV virions (**E**), and Dane particle (**F**). Details concerning the analysis of each component are given in the diagram as well as in the Section 2. RU, relative unit, normalized to D0 + dox. ns: *p*-value > 0.05; *: *p*-value ≤ 0.05; **: *p*-value ≤ 0.01; ***: *p*-value ≤ 0.001. ‡: ~6.1 × 10^6^ HDV RNA copies/mL; §: 5.3 × 10^5^ HBV DNA copies/mL.

A kinetic of HDV expression was conducted with HepG2BD cells expended by regular passages (every 3 to 4 days). Cells were pretreated with dox and cultured for 42 days +/− dox (Figure 2). When dox was withdrawn, the launch in HDV RNA replication became detectable from day 10 post-removal (Figure 2A,B). Strand-specific detection of the antigenomic HDV RNA followed a similar trend (Figure S3). Concomitantly, the transcription of 3.5 kb RNAs (Figure 2C) and the secretion of HBV virions (Figure 2F) decreased. Subviral particles (SVP) secretion was overall unaltered (Figure 2D). Secretion of HDV virions increased starting at day 14 post-removal of dox and reached a plateau by day 17. In the presence of dox, intracellular HDV RNA was significantly suppressed, in line with a tight silencing of the transcription of genomic HDV RNA through the repression of the Tet-Off promoter. 

**Figure 3:**
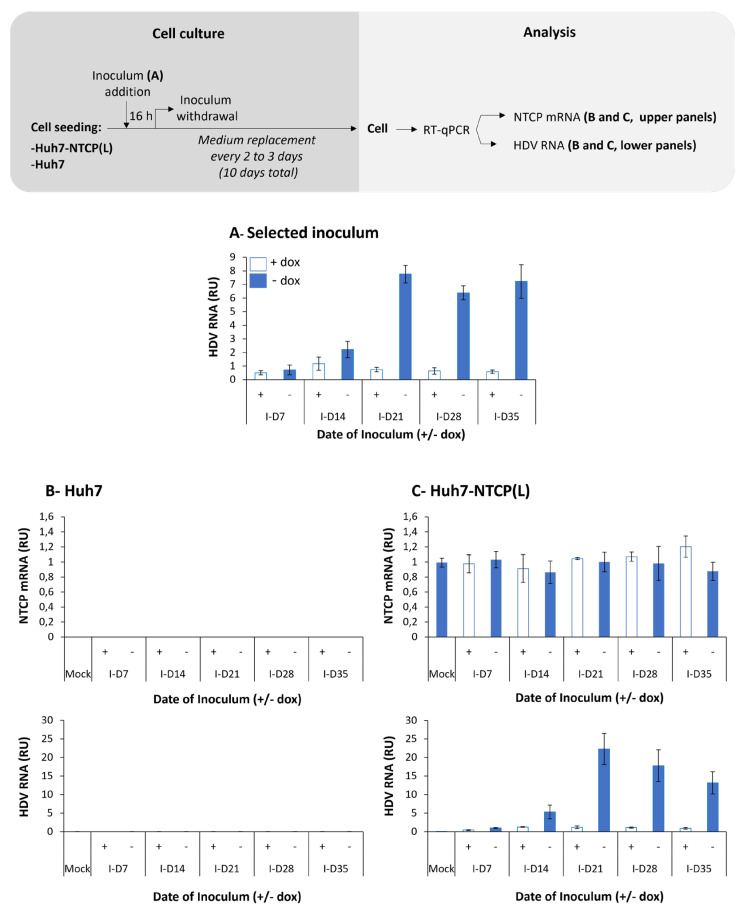
Infectivity of supernatant from HepG2BD cells. Selected HDV RNA-containing supernatants (**A**) from experiment described in Figure 2 were used to inoculate Huh7 (negative control) and Huh7-NTCP(L) as depicted in the diagram. At day 10 post-inoculation, cells were lysed and NTCP mRNA as well as HDV RNA were quantified in parallel in both Huh7 (**B**) and Huh7-NTCP(L) (**C**) by RT-qPCR. RU, relative unit, normalized to mock.

Infectivity of HDV virions produced by HepG2BD cells in Figure 2 was monitored after inoculation of Huh7-NTCP cells (see Section 2) with equal volumes of selected supernatants collected between days 7 and 35 of the kinetic experiment (Figure 3A). Huh7 cells were used as the negative control (Figure 3B). Levels of intracellular HDV RNA at 10 days post-inoculation showed that Huh7-NTCP cells were susceptible to infection with HDV virions produced by the HepG2BD cells (Figure 3C, lower panel). 

### 3.3. Ongoing HDV Replication in HepG2BD Is Efficiently Inhibited by the Addition of Doxycycline

**Figure 4:**
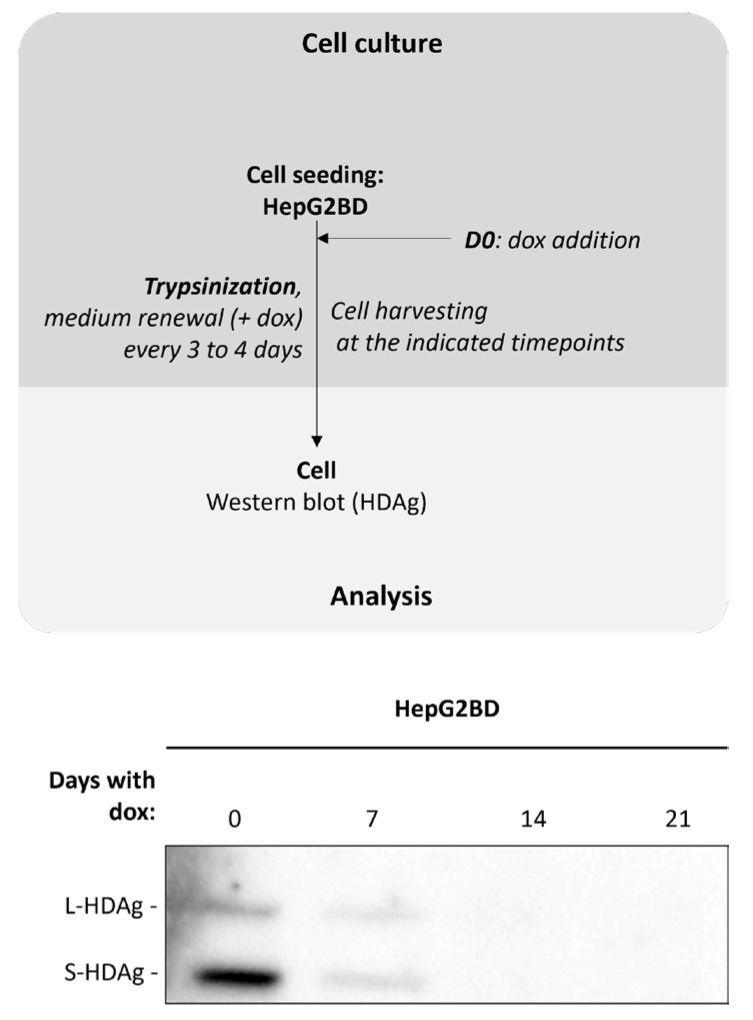
Inhibition of HDV replication by addition of doxycycline in HepG2BD. Cells were subjected to dox treatment for up to 21 days as depicted in the diagram and analyzed by Western blot for their ability to synthesize HDAg isoforms at each of the indicated timepoints.

We sought to evaluate the effect of dox reintroduction on viral replication. To this end, cells were cultured in dox-free medium for 14 days prior to regular passages in the presence of the 2 µg/mL dox for 21 days. Results show a significant decrease in intracellular HDAg at day 7 in dox-treated cells, with only trace amounts at day 14 (Figure 4). 

### 3.4. Quiescence of HepG2BD Cells Enhances HDV Production

**Figure 5:**
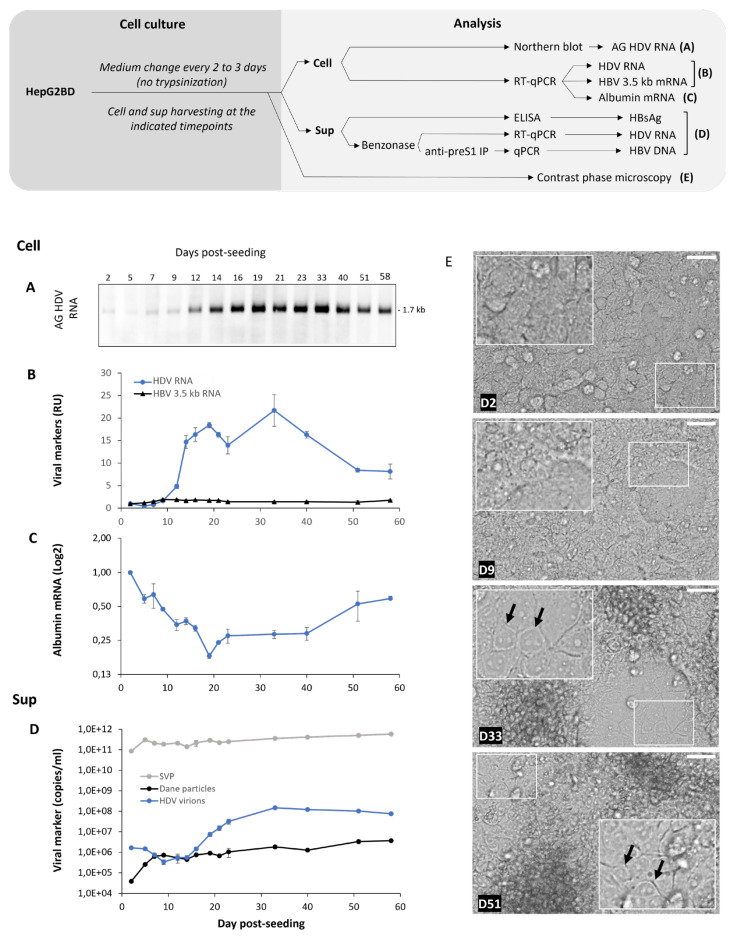
Effect of HepG2BD cell line prolonged quiescence on HDV and HBV replication, intracellular albumin RNA, and viral secretion: 58 days course. The conducted experiment is depicted in the diagram and aimed to assess the effect of non-trypsinization of the cells on HDV and HBV viral lifecycles as well as on albumin mRNA, a liver function marker. (**A**) Northern blot analysis of cellular antigenomic HDV RNA. (**B**) Cellular HDV RNA and 3.5 kb HBV mRNA quantifications. (**C**) Albumin mRNA quantifications. (**D**) HBsAg, HDV RNA, and HBV DNA monitoring in the supernatant. (**E**) Cells were observed by contrast phase microscopy at different timepoints post-seeding as indicated for each picture. Arrows point toward HepG2BD cells with hepatocyte-like morphology. Details about the analysis are depicted in the diagram as well as in the Section 2. Scale bars: 50 µM. RU, relative unit, normalized to D2. AG, antigenomic.

We conducted a kinetic experiment of HepG2BD cultured with no passage for up to 58 days (Figure 5). Intracellular HDV RNA was detected at increasing levels until day 33 (Figure 5A,B) after which a slight decrease occurred. Secretion of HDV virions increased starting at day 19 and peaked at day 33 (~1.5 × 10^8^ ge/mL). The secretion of HDV virions did not correlate with a surge of SVP secretion (Figure 5D), indicating that as opposed to the Huh7-END, HBV envelope proteins are not a limiting factor for HDV secretion by HepG2BD cells. 

Intracellular HBV 3.5 kb RNA increased until day 9 and remained constant for the duration of the follow-up (Figure 5B). Secretion of HBV virions increased at the late stage of culture and peaked at day 58 (~3.7 × 10^6^ ge/mL) when secretion of HDV virions had started to decrease. 

The levels of mRNA coding for liver-specific albumin decreased from day 1 to 19, then rebound between days 19 and 58 (Figure 5C). The increase in HBV virions and SVPs secretion (Figure 5D) followed the same trend as the rebound in albumin (Figure 5C). The differentiation of HepG2BD cells is suggested by the observation of a morphological change toward “hepatocyte-like” cells at day 58 in culture (Figure 5E).

**Figure 6:**
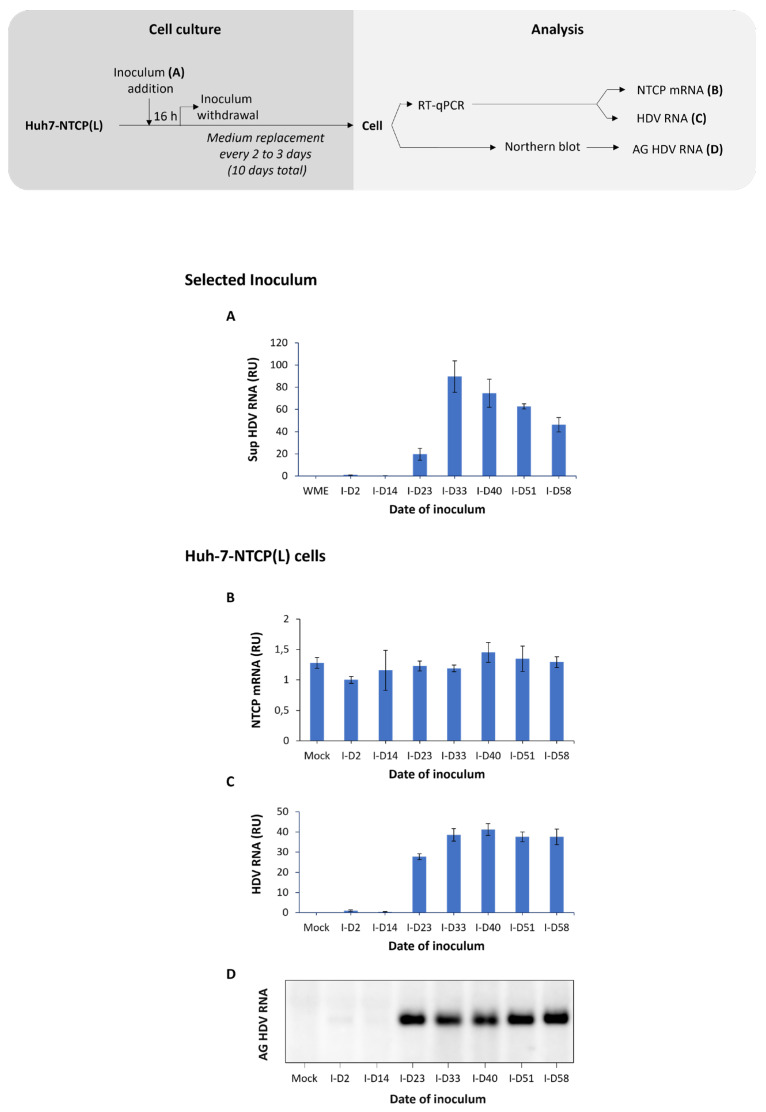
Infectivity of HDV virions produced from quiescent HepG2BD. Selected HDV RNA-containing supernatants (**A**) from experiment described in Figure 5 were used to inoculate Huh7-NTCP(L), as depicted in the diagram. At day 10 post-inoculation, cells were lysed and the levels of NTCP mRNA (**B**), HDV RNA (**C**), and antigenomic HDV RNA (**D**) were estimated. RU, relative unit, normalized to I-D2. AG, antigenomic.

Infectivity of HDV particles collected from the experiments in Figure 5 was tested by inoculation of Huh7-NTCP(L) cells. Intracellular levels of HDV RNA demonstrate that HDV virions were infectious (Figure 6). 

Altogether, the results show that quiescence of HepG2BD cells leads to a significant increased secretion of HDV virions (maximal concentration of ~1.5 × 10^8^ RNA copies/mL of supernatant), as well as HBV virions (maximal concentration of ~3.7 × 10^6^ DNA copies/mL in Figure 5D). These increases are accompanied by a very distinct trend of albumin mRNA concentration, as well as a morphological change to “hepatocyte-like” cells.

### 3.5. Effect of Culture Conditions on the Rate of HDV-Positive Cells

**Figure 7:**
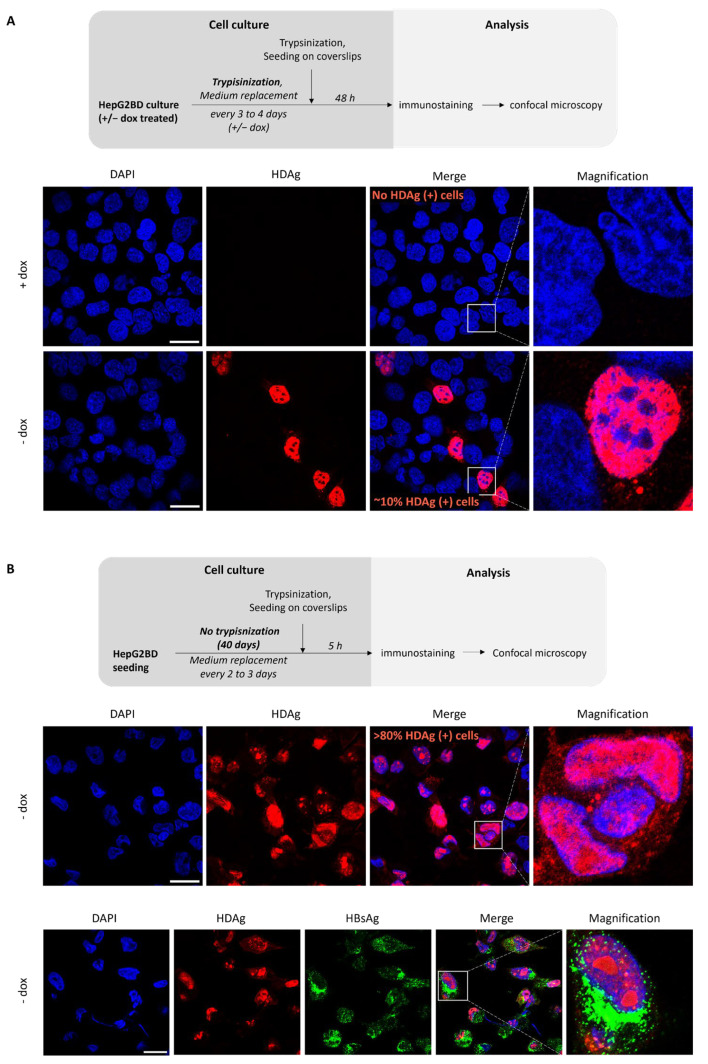
Effect of culture pattern and presence of doxycycline on the rate of HepG2BD cells actively replicating HDV. (**A**) As depicted in the diagram, HepG2BD cells were regularly trypsinized in the presence or absence of dox, prior to seeding on glass coverslip for 48 h, and fixation with PFA. Cells were then stained with DAPI (blue) and with an anti-HDAg antibody (red). (**B**) HepG2BD cells were seeded and maintained in collagen-treated wells in the absence of dox for 40 days. Cells were then trypsinized and seeded on coverslips for 5 h, fixed with PFA, and stained with DAPI (blue), and an anti-HDAg antibody (red). An additional immunostaining of HBsAg (green) was conducted in the lower set of pictures. Scale bars: 25 µM.

HepG2BD cells were either (i) regularly passaged to favor the proportion of actively dividing cells or (ii) maintained at confluency for 40 days to reach optimum quiescence/differentiation. In actively dividing cultures in the presence of dox (Figure 7A), no HDAg-positive cells were observed, while approximately 10% of actively dividing cells harvested at day 3 post-seeding demonstrated detectable levels of fluorescence in the absence of dox. This result shows the ability of dox to tightly regulate the HDV RNA and HDAg proteins.

When the same experiment was conducted on quiescent cells (Figure 7B), virtually all cells were positive for HDAg. This result clearly demonstrates that the low proportion of HDAg-positive cells in Figure 7A was not due to a lack of HepG2BD clonality and suggest that active replication is initiated in quiescent cells.

### 3.6. HepG2BD as a Tool for Antivirals Analysis

**Figure 8:**
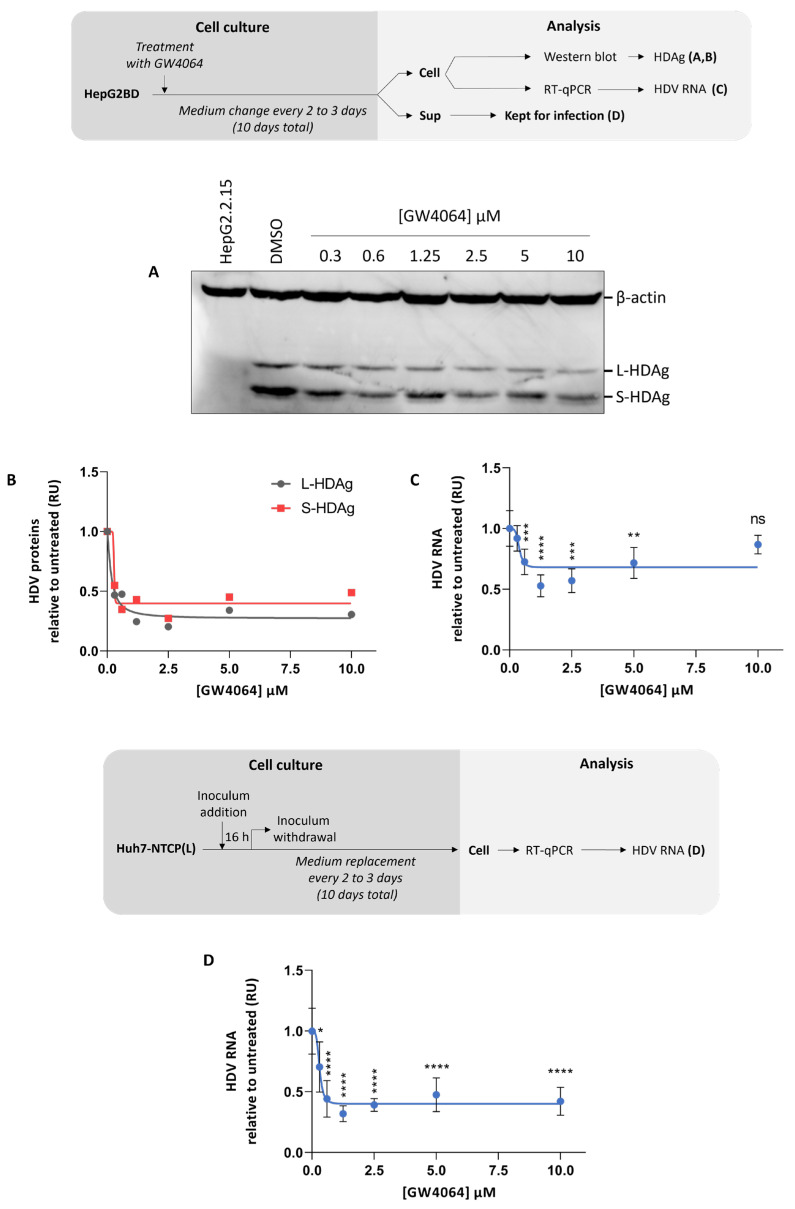
Antiviral effect of FXR agonist GW4064 on HDV in the HepG2BD cell line. As depicted in the diagram, cells were treated with various concentrations of GW4064. At 10 days post-treatment, cells and supernatant were harvested. Cellular total proteins were analyzed for the presence of HDAg isoforms by Western blot (**A**). Densitometry analyses from (**A**) are presented as ratios of S-HDAg or L-HDAg normalized to levels of β-actin (**B**). Intracellular HDV RNA (**C**) were quantified. As depicted in the diagram, Huh7-NTCP(L) cells were infected with harvested supernatants and cultured for 10 days. HDV RNA were quantified by RT-qPCR (**D**). RU, relative unit, normalized to mock. ns: *p*-value > 0.05; *: *p*-value ≤ 0.05; **: *p*-value ≤ 0.01; ***: *p*-value ≤ 0.001; ****: *p*-value ≤ 0.0001.

Recently, the FXR agonist GW4064 was reported as a potent inhibitor of HDV replication in HepaRG and Primary Human Hepatocytes (PHH) [28]. To analyze the effect of GW4064 on HDV replication, we treated HepG2BD cells with an increasing concentration of GW4064 for 10 days. HDV proteins were then extracted and analyzed by Western blot (Figure 8A), and the densitometry ratios of S- or L-HDAg/β-actin are presented (Figure 8B). Both the Western blot and densitometry ratio show a dose-response antiviral effect of the GW4064. Of note, the inhibition of the L-HDAg (75%) was slightly higher than of the S-HDAg (60%). Total intracellular RNAs were also harvested and analyzed by RT-qPCR (Figure 8C), demonstrating a weaker dose-dependent antiviral effect (35%). Overall, we observed that GW4064 has a greater inhibitory effect on HDV proteins than on HDV intracellular RNA (compare Figure 8B,C). To assess the amount of infectious HDV virions produced under GW4064 treatment, cell culture supernatants were collected and used to infect naive Huh7-NTCP(L) cells. At 10 days post-infection, total intracellular HDV RNAs were analyzed by RT-qPCR. As shown in Figure 8D, a dose-dependent inhibition, up to 62%, was observed.

## 4. Discussion

Recently, the proportion of HDV coinfection in the population of HBV carriers was evaluated up to 70 million worldwide [3]. Although HBV mono-infected patients benefit from efficient antiviral molecules (NUCs) that reduce viremia by inhibiting the viral DNA polymerase [29], these drugs are of very limited benefit to HDV carriers, due to their lack of impact on both HDV RNA replication and HBV envelope proteins synthesis [30]. To this date, the only available treatment for HDV-infected patients is based on PEG-IFN or bulevirtide [6,7]. Although new treatments are in development [31], there is a need to identify/characterize new HDV-specific therapeutic compounds, and hence a necessity for the development of models allowing chronic concomitant replication of HBV and HDV. 

The HepG2BD cell line described here is unique in being permissive for replication of both HBV and HDV. Moreover, while the replication of HBV is constitutive in this cell line, the replication of HDV RNA is regulated by the presence/absence of dox. This model may thus serve as an in vitro mimic of HDV superinfection in vivo.

As opposed to previously reported cell lines that relied on random integration of HDV cDNA into cell DNA [13,14,15] for synthesis and replication of HDV RNA, our model was engineered using the CRISPR-Cas9 technology for insertion of only one copy per chromosome 19. Indeed, HDV cDNA insertion into the AAVS1 safe harbor maximizes the control of induction/repression while minimizing the risk of off-target alteration. This experimental approach was combined with the use of a Tet-Off promoter for transcription of HDV RNA from the integrated DNA template. Our results demonstrate that HDV RNA replication in HepG2BD cells is indeed tightly turned off in the presence of dox. 

HepG2BD cells could be proliferated for long periods (>6 months) without loss of inducible HDV replication and secretion of infectious virions. Cells could develop a hepatocyte-like phenotype associated with increased HDV replication (>80% positive cells). Strikingly, resting HepG2BD cells presented an altered albumin mRNA synthesis, a morphological change to hepatocyte-like phenotype, and a significant increase in HDV virions secretion, culminating at ~1.5 × 10^8^ ge/mL. HepG2BD cells maintained at a differentiated stage may thus serve as an in vitro model of an inactive HDV/HBV coinfection in vivo, while actively dividing cells might mimic an active clinical hepatitis with an elevated hepatocyte turnover. In the absence of dox, around 10% of proliferating HepG2BD cells were positive for nuclear HDAg, as opposed to more than 80% in resting cells. This latter observation was taken as further evidence that the HepG2BD cell line is clonal. The lack of a strong intracellular co-localization of HDAg and HBsAg in resting cells may reflect the natural propensity of HBV envelope proteins for self-assembly into SVPs.

Contrary to what was reported for the Huh7-2C8D cells, we did not observe a switch over time in the S-HDAg/L-HDAg proteins ratio either in actively dividing HepG2BD cells (Figure 2) or in resting cells (Figure S4). In a typical HDV replication cycle, the incoming HDV genome (coding for the S-HDAg protein) is replicated in the cell nucleus but in the course of genome replication there is an increasing level of genomes coding for L-HDAg that leads to HDV virions assembly and to a shutdown of replication [15,32,33]. The fact that the S-HDAg/L-HDAg ratio remains >1 in HepG2BD cells is likely due to the replenishment of cells with de novo, non-edited, i.e., S-HDAg-coding genome transcribed from the integrated DNA template, allowing long-term viral replication of viral RNA.

It is known that HDV replication could decrease HBV viremia in patients [34]. Importantly, a recent study described an HDV-induced interferon-dependent and -independent inhibition of HBV in human hepatocytes [35]. Here, the kinetics of HBV/HDV replication in actively dividing (Figure 2) and resting (Figure 5) HepG2BD cells present evidence of an interplay between HBV and HDV. Indeed, intracellular HBV pgRNA and HBV virions secretion were repeatedly suppressed when HDV RNA replication increased. While additional experiments are needed to further document this interplay, the HepG2BD cell line appears suitable to pursue the characterization of the mechanisms involved in the HBV/HDV dynamics. It should allow for the in vitro recapitulation of patterns observed in chronic HDV infection, in which periods of HBV- or HDV-dominance alternate with each other throughout the course of infection.

In order to validate the usefulness of the HepG2BD cells as a tool for antiviral analysis, the FXR agonist GW4064 was tested in a dose-dependent manner (Figure 8). Intracellular anti-HDV activities obtained in HepG2BD treated with GW4064 were comparable to those obtained in PHH by Legrand and colleagues but significantly weaker than those obtained in HepaRG [28]. A possible explanation is that in HepG2BD, HDV replication is more uniform and remains stable throughout the experiment, whereas in HepaRG, HDV replication is weaker and transient. Regarding infection results, the anti-HDV antiviral effect seems lower in HepG2BD compared to HepaRG [28]. These differences could also be explained by a differential in HDV replication between HepG2BD and HepaRG as well as by the different cells used for infection (Huh7-5-NTCP vs. Huh7-NTCP(L)). In future studies, it would be interesting to evaluate simultaneously the antiviral activities of compounds on the replication of both HBV and HDV. HepG2BD could allow antiviral assays in a context of chronic co-infection (no dox), but also in the context of superinfection with active HBV replication in the presence of dox, followed by removal of dox to activate HDV replication.

In summary, the HepG2BD cell line represents a unique, innovative, and versatile in vitro model for HBV/HDV coinfection, which is easily amenable to high throughput screening and the characterization of antivirals. Proliferating HepG2BD cells may serve as an in vitro model of chronically coinfected patients suffering from an active hepatitis associated with elevated liver-turnover, while resting/differentiated cells would rather reflect an inactive pattern of coinfection. The HepG2BD cell line is also expected to help in our understanding of the dynamic interplay between the HBV and HDV lifecycles, and in identifying cellular factors involved in dividing or resting cells.

## Data Availability

Data are available upon request from the corresponding author.

## Supplementary Materials

The following supporting information can be downloaded at: https://www.mdpi.com/article/10.3390/v16040532/s1, Figure S1: Use of the chromosomal-driven expression of the resistance gene for the selection/characterization of HDV-expressing colonies; Figure S2: Verification of proper insertion of the donor sequence in the AAVS1 safe harbor by touchdown PCR; Figure S3: Quantification of antigenomic HDV RNA through strand-specific RT-qPCR; Figure S4: Quantification of S-HDAg/L-HDAg ratio over time.

## Abbreviations

HBV: hepatitis B virus; HDV, hepatitis Delta virus; AAAVS1, adeno-associated virus safe harbor integration site 1; HCC, hepatocellular carcinoma; Dox, doxycycline; Puro, puromycin; PEI, polyethylenimine; NTCP, sodium taurocholate co-transporting polypeptide; HBsAg, hepatitis B surface antigen; L-HDAg, hepatitis delta large antigen; S-HDAg, hepatitis delta small antigen; FXR, farnesoid X receptor; SVP, subviral particles.
