# Supplementary material for: HepG2BD: A Novel and Versatile Cell Line with Inducible HDV Replication and Constitutive HBV Expression
Source: Viruses. 2024 Mar 29;16(4):532. doi: 10.3390/v16040532 (PMC11053718; doi:10.3390/v16040532)
Supplement: Supplementary file 1 [file viruses-16-00532-s001.zip › viruses-2916414-supplementary.pdf]

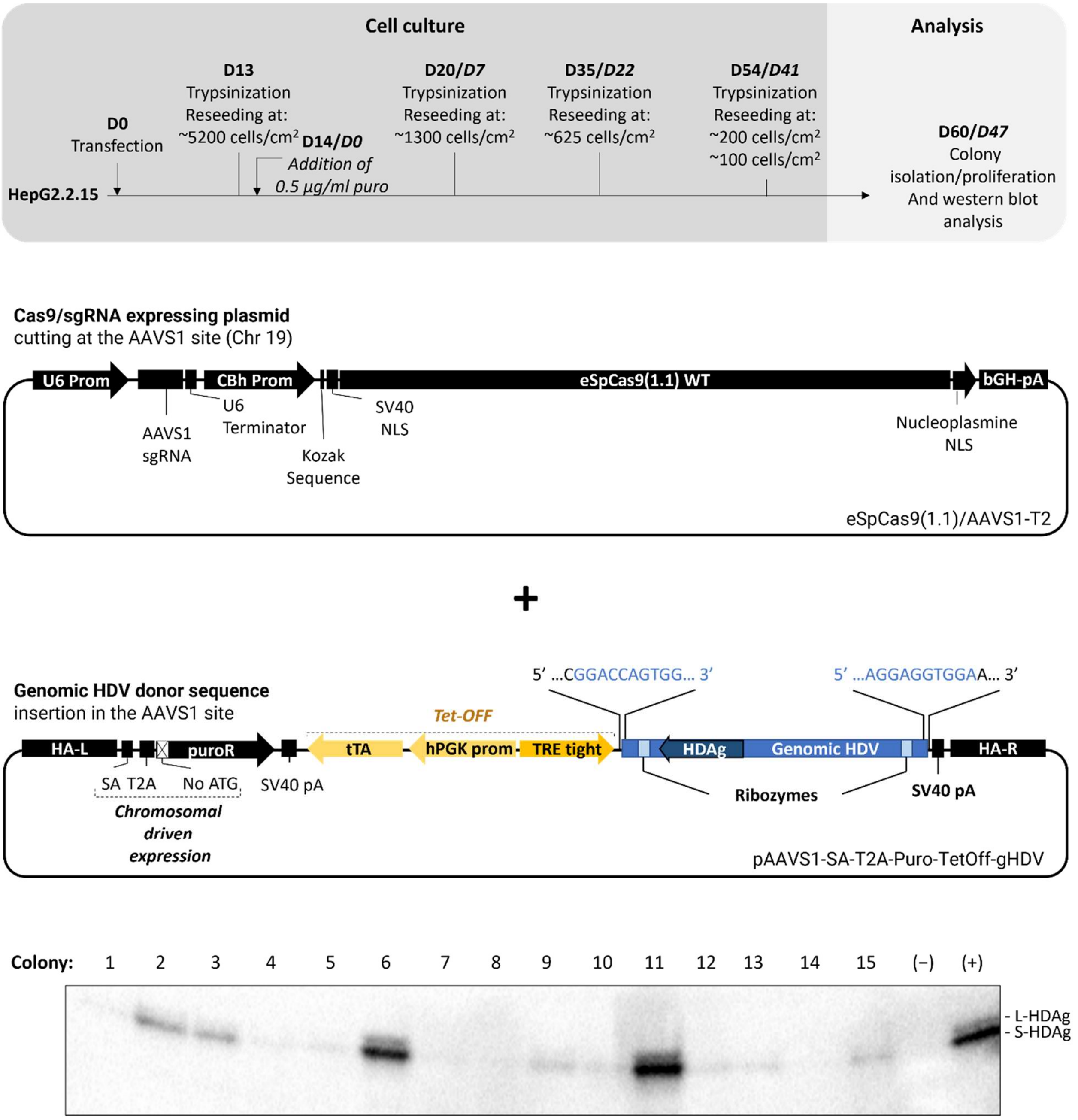

Figure S1. Use of the chromosomal-driven expression of the resistance gene for the selection/characterization of HDV expressing colonies. HepG2.2.15 cells were co-transfected with the indicated plasmids. Following culture was conducted as depicted in the diagram. At day 60 post-transfection (day 47 post-initiation of puro treatment), colonies were isolated, proliferated, and tested for the presence of HDAg isoforms by western blot. (-), negative control using non transfected HepG2.2.15 cells; (+), positive control using the lysate of remaining cells after colony harvesting. Numbers in italic indicate time post-addition of puro.

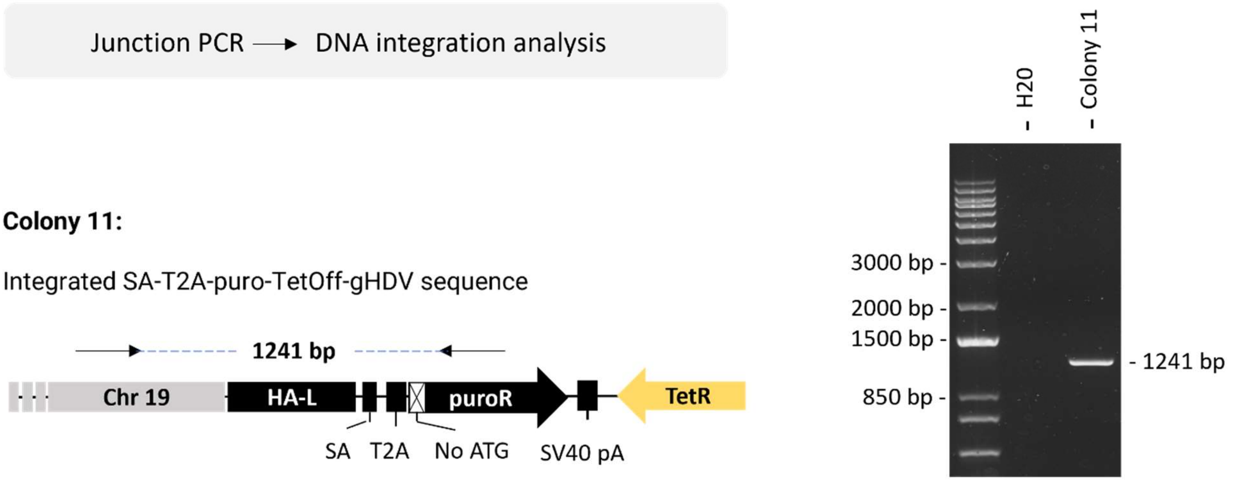

Figure S2. Verification of proper insertion of the donor sequence in the AAVS1 safe harbor by touchdown PCR. To make sure that the transgene was properly integrated in the AAVS1 genome safe harbor, we conducted a touchdown PCR using the iProof™ High-Fidelity DNA Polymerase (Bio-rad). The forward primer (5′-ccctggccattgtcactttg-3′) was designed to anneal on the chromosomal sequence, 5′ to the left homology arm (HA-L), while the reverse primer (5′-gagttcttgcagctcggtgac-3′) was designed to anneal on the puroR sequence. Cycling was as follows: initial denaturation for 3 min at 98°C, followed by 15 cycles of touch down amplification. Each cycle begun with 10 sec at 98°C followed by initial annealing for 10 sec at 72°C with a decrease by 0.5°C at each cycle. Elongation was carried out at 72°C for 1 min at each cycle. Regular PCR was then conducted with 25 five cycles with denaturation at 98°C for 10 sec, 64°C for 10 sec, and 72°C for 1 min. ultimately a final elongation step of 10 min at 72°C was conducted and PCR products were migrated on a 1% agarose gel.

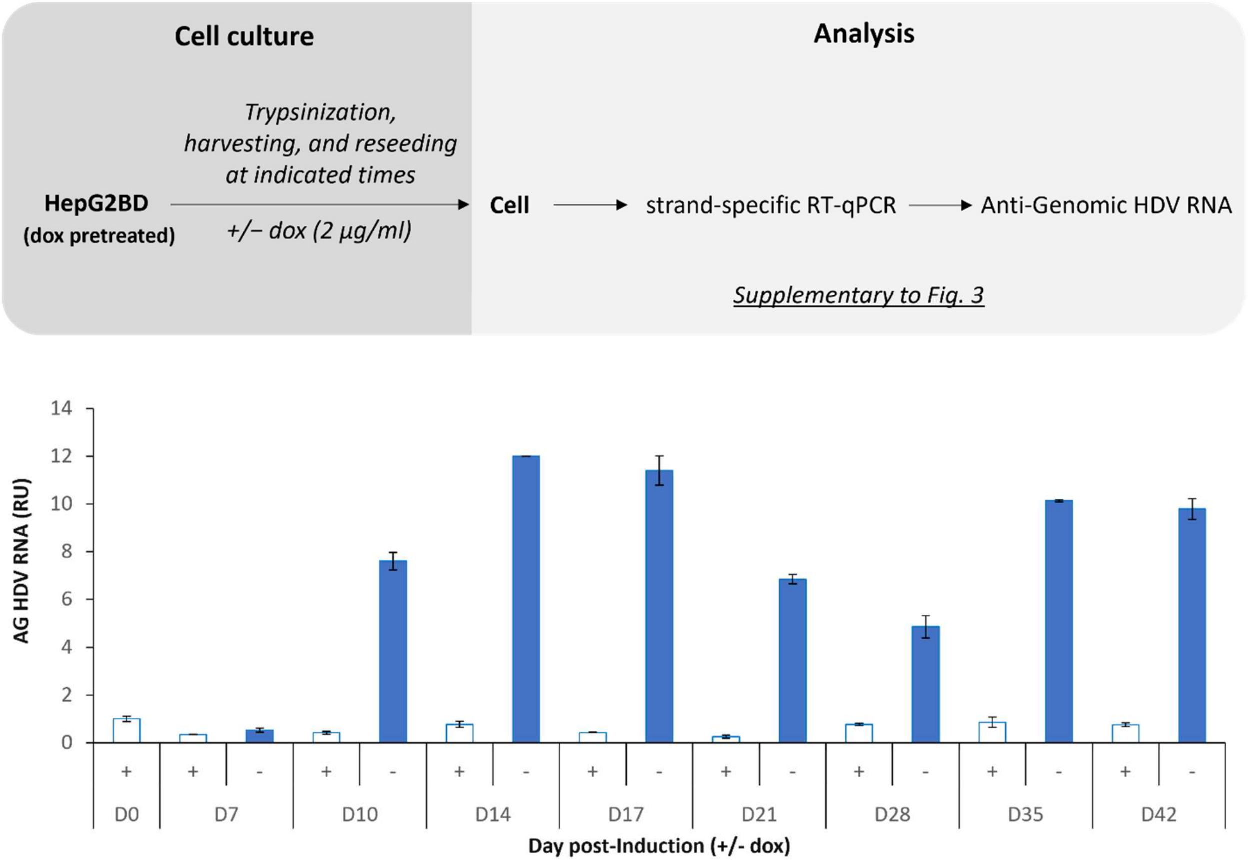

Figure S3. Quantification of antigenomic HDV RNA through strand-specific RT-qPCR. This experiment was conducted to monitor the presence of antigenomic RNA, which is a marker for rolling circle replication of the HDV genome, in samples from Fig. 3A and B. RNA extraction was conducted as described in the method section. Reverse transcription for antigenomic HDV RNA quantification was conducted as described in the section 2 with the following alterations: an elongation temperature of 55°C and the replacement of random hexamers with a 5'-tggacgtgcgtcctcct-3′ primer. The following Taqman qPCR was conducted as described in the section 2. RU, relative units, relative to D0.

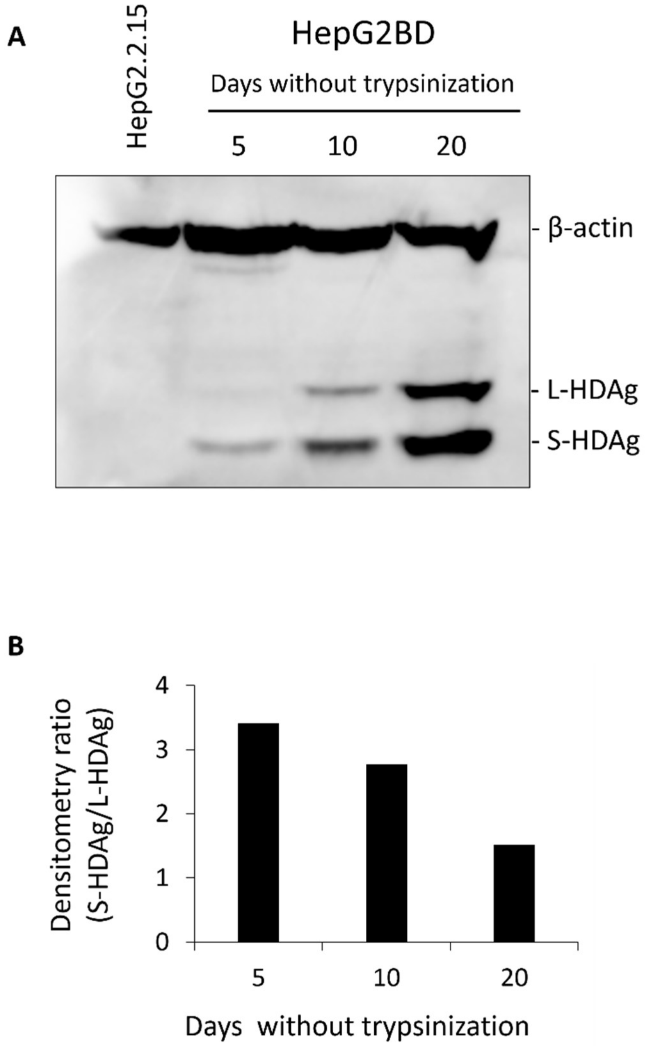

Figure S4. Quantification of S-HDAg/L-HDAg ratio overtime. This experiment was conducted to monitor the ratio of S-HDAg produced over L-HDAg overtime. HepG2BD were cultured without trypsinization for the indicated number of days. (**A**) Western blot was performed as described in the section 2. (**B**) Densitometry ratios of S-HDAg/L-HDAg from western blot in (**A**).
